# Experiences of microdosing psychedelics in an attempt to support wellbeing and mental health

**DOI:** 10.1186/s12888-023-04628-9

**Published:** 2023-03-14

**Authors:** Rebecca S. Ryan, Alex Copello, Andrew P. Fox

**Affiliations:** School of Psychology, University of Birmingham, Edgbaston, Birmingham, B15 2TT UK

**Keywords:** Microdosing, Psychedelics, Psilocybin, LSD, Mental health, Wellbeing

## Abstract

**Background:**

Microdosing psychedelic drugs is a growing phenomenon, but little is known about the experiences surrounding this. Research broadly suggests that people may use psychedelics in an attempt to self-medicate for mental health and wellbeing. However, the precise details, rationale and meaning of such attempts remains unclear, and would benefit from clarification, using tailored experiential methods. This research therefore aimed to explore the way that users make sense of microdosing psychedelics, with a particular focus on the experience of any perceived mental health or wellbeing changes.

**Method:**

Participants were recruited via websites and online forums. An internet text-based, semi-structured interview was conducted anonymously with 13 participants regarding their experiences of microdosing psychedelic drugs. Interpretive Phenomenological Analysis was used to analyse the transcripts.

**Results:**

Three superordinate themes were identified through the interviews: 1) Seeking a solution: Agency and rationale; 2) Microdosers as scientists; 3) Catalysing desirable and beneficial effects.

**Conclusions:**

All participants approached microdosing methodically and with purpose. Participants reported that they had experienced beneficial effects of microdosing on their mental health, alongside cognitive, physical and social changes. By microdosing, participants reported that they had supported their own mental health and wellbeing, with microdosing described as a catalyst to achieving their aims in this area. This study provided additional knowledge and understanding of the experience, rationale and personal meaning of the microdosing phenomenon which can be used to inform future investigations in the areas of psychedelic use and mental health.

**Supplementary Information:**

The online version contains supplementary material available at 10.1186/s12888-023-04628-9.

## Background

In the 1950s there was growing interest in the therapeutic use of LSD (lysergic acid diethylamide), as well as other classic hallucinogens, such as mescaline [6]. Hallucinogenic drugs were considered for therapeutic benefits in the treatment of mood disorders, obsessive–compulsive disorders and addiction [20]. However, by the 1970s, many western countries enforced the prohibition of psychedelic drugs due to their association as drugs of abuse and danger [24]. More recently, despite these substances still being illegal in most countries, research has once again recommenced. For example, more recent controlled trials have used psychedelic drugs such as LSD and psilocybin (or ‘magic mushrooms’) with human participants to explore the social, psychological and biological effects. One such study by Carhart-Harris et al. [5] found that after two oral doses of psilocybin, there were significant reductions in depressive symptoms in 20 participants and that symptom improvement remained six months post-treatment.

Reports indicate that people are beginning to experiment with very small doses of psychedelics, such as ‘microdosing’—ingesting a very low dose of a psychedelic substance, usually in a routined schedule [9]. The growing popularity of microdosing is illustrated in news coverage and in active online communities of microdosers, with large numbers of individuals reportedly experimenting with microdosing in the hope of psychological and wellbeing benefits [18]. Currently, research into microdosing is still in the early stages. Some studies have begun to investigate this phenomenon such as Prochazkova et al. [19], an open-label study investigating truffles, Yanakieva et al. [27] a double-blind placebo-controlled study looking at microdosing LSD and time-perception, and Bershad et al. [4] which was a controlled laboratory setting study also looking at effects of microdosing LSD.

In a study by Polito and Stevenson [18], the authors found that short-term microdosing led to an effect across a number of psychological variables, but these effects diminished over subsequent days. Over a longer period, there was evidence of improvements in depression and stress, altered attentional capacities and increased neuroticism. However, the variables that participants reported that they had expected to change showed no evidence of change [18]. In a recent study by Kaertner et al. [13], web-based surveys with participants who were planning on microdosing showed increased psychological wellbeing, improved emotional stability, and reduction in depressive and anxious symptoms. However, the expectancy scores at baseline and then the following improvements were indicative of a placebo effect.

An online study by Hutten et al. [10] found that the main motivation for participants to microdose was performance enhancement (37%), mood enhancement (29%) and relief of symptoms (14%). A second online study by Hutten et al. [11] showed that microdosing was more responsive in alleviating symptoms from a number of mental or physiological difficulties, compared to conventional treatments. However, the effect of microdosing was reduced compared to that of full psychedelic doses.

A further online study found that microdosers self-reported reduced levels of dysfunctional attitudes (towards themselves, other and the world) and negative emotions, with increased self-reported wisdom, open-mindedness and creativity, relative to people who had never microdosed [2]. As part of this study, Anderson et al. [1] developed a codebook of microdosing and noted that microdosers reported beneficial outcomes in improved mood, focus and creativity. In terms of challenging outcomes, physiological discomfort and increased anxiety were highlighted.

Johnstad [12] interviewed people online who had microdosed and respondents described benefits to mental health, especially on symptoms of depression and anxiety, as well as improved energy, cognition, and creativity, and few reported adverse effects. Webb, Copes and Hendricks [25] interviewed 30 people and found participants rationalised microdosing in a functional and considered manner that separated themselves and those who use drugs recreationally and hedonistically. Lea et al. [14] completed a content analysis of microdosing discussions on Reddit (an online forum), and found that those who were involved in these discussions were motivated to microdose to improve their mental health, wellbeing and cognitive performance. For some, microdosing had achieved or exceeded expectations for those posting in the discussions. However, some also reported no effect or increased anxiety whilst microdosing. A further online survey with 1102 respondents found that respondents were microdosing to help with depression (21%), anxiety (7%), other mental disorders (9%) and for substance cessation or reduction (2%) [15].

Alongside growing media interest and community reports of microdosing psychedelics, academic research indicates that people may be using psychedelic drugs in an attempt to self-medicate for mental health purposes [7, 17] and/or with a motivation to improve wellbeing [12, 13]. However, most research conducted on microdosing such as Yanakieva has not explored such variables [27]. Therefore, clarification is required, and there is a need to further understand the outcomes of the research thus far, and the claims of media and anecdotal community reports like those on Reddit. Building on existing research, a more specific understanding of the reasons people use these substances for their mental health and/or wellbeing is required to elaborate on the broader explorations developed so far. The present study sought to explore this, taking a neutral stance to the use of microdosing psychedelics – neither condoning nor condemning—so that all aspects of the experience could be explored. The aim was to investigate the experience of microdosing psychedelic drugs with a particular focus on the perceived effects on participants’ mental health and/or overall sense of wellbeing. In order to meet the aim of this study, and to allow the study to be explorative in nature, semi-structured interviews of a focused sample facilitated flexibility in exploring in detail what is important to the participants and how they made sense of this. This study was novel in that it used IPA (Interpretative Phenomenological Analysis) as an analytical tool particularly designed to develop an understanding of people’s experiences, by exploring meaning making and phenomenology. This is in comparison to other studies, for example Hutten et al. [10, 11] and Kaertner et al. [13], who used online questionnaires and web-based surveys, and Johnstad [12] who used Thematic Analysis. To ensure anonymity of participants the study was designed in a way that enabled those taking part to remain anonymous whilst being able to complete a comprehensive interview.

## Method

### Definitions

Microdosing means ingesting a very low dose of a psychedelic substance [9]. As defined by Nichols [17], psychedelics were categorised as the classic serotonergic hallucinogens which have a similar method of working, such as LSD, psilocybin, DMT and mescaline.

There are a number of definitions for wellbeing due to it being a multifactorial concept lacking a singular definition [8]. Some have suggested that wellbeing can be divided into objective and subjective measures [21, 23]; as this study explored the subjective experience of participants more broadly, the concepts of wellbeing and mental health were used to allow participants to describe their own experiences of these areas. Participants did not have to have a diagnosed mental health illness in order to participate in the study. They were required to have experienced difficulties with mental health and/or wellbeing based on their perception and understanding of these definitions, and were microdosing in an attempt to support their perceived difficulties.

### Recruitment of participants

**Fig. 1:**
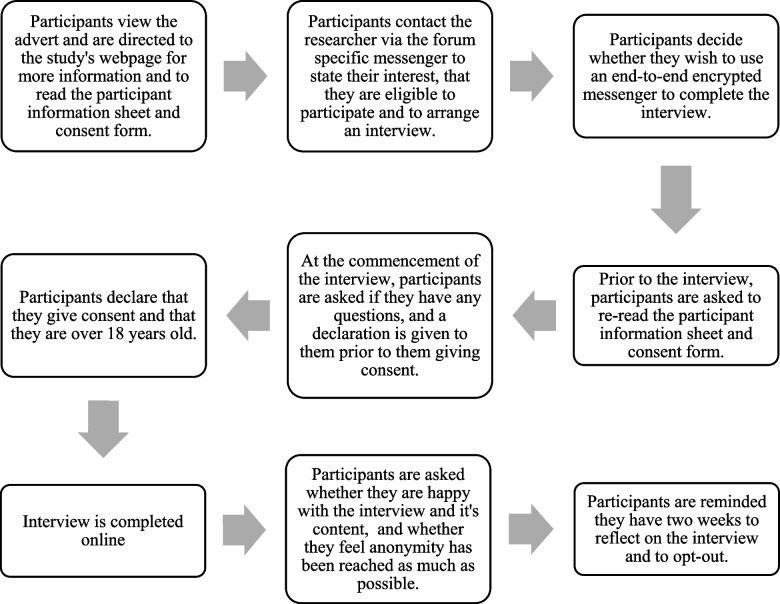
Procedure for the study

Purposive sampling was used to recruit participants who had experience of microdosing psychedelic drugs (see Fig. 1). Adverts for the study were posted on internet fora such as Reddit, The Shroomery.org, and Bluelight.org, however, the latter two did not produce significant recruitment responses. These internet fora were chosen due to their use in other research, such as Carhart-Harris and Nutt [7], Johnstad [12] and Polito and Stevenson [18]. Furthermore, they all had forum-specific messengers that could be used for participants to contact the researcher. Participants approached the researcher via the forum’s instant messenger, to say they were interested in participating in the study and as per the advert, they were asked to do so using a non-identifying username. The researcher did not approach anyone on these internet fora to ask them to participate; participants responded directly to the adverts only. Once enough data has been collected, anyone who subsequently approached the research was informed that recruitment had closed.

Inclusion and exclusion criteria are detailed in Table 1. Due to the study focussing on exploring microdosing in terms of wellbeing and mental health, those who reported engaging in microdosing for any other purpose were excluded from the study.

**Table 1:** Inclusion and exclusion criteria

**Inclusion Criteria**	**Exclusion Criteria**
Those aged 18 and over	If currently microdosing to cope with substance use problems/withdrawal
Those with experience of being in a regime of microdosing a classic psychedelic drug to support their mental health and/or wellbeing	If microdosing for any other purpose e.g. recreational purposes
Those able to confidently read and write in English	

### Anonymity and confidentiality

Given that the reported study involved interviewing participants about their illegal drug use via forum-specific instant messengers, it was a prerequisite of the study that if an individual was to give informed consent, then they had to use a username which was not linked to their real name nor identity. As participants were mostly recruited via Reddit and participants used non-identifying usernames, there were no links to any social media profiles which could have revealed their identity. There was no collection of identifiable information such as email addresses or telephone numbers. As an added precaution for anonymity purposes, aside from using the forum-specific instant messengers, participants were given the option to sign up to an end-to-end encrypted online instant messenger service for the interview. This meant that the online interview details could only be seen between researcher and participant and no other body. This method appeared preferable to participants, and had the added value that the interview would be destroyed by messenger service following completion.

### Participants

Table 2 provides an overview of each participant using an anonymised name. In total 13 participants were recruited for the study, which is a suitable number to complete IPA due to the idiographic focus on lived experience [22]. Out of the 13 participants, 10 were male and three were female, with an average age of 34.9 years old. All participants had been educated to college level or above, with over 50% of participants having gone on to complete an undergraduate degree or postgraduate degree. All participants except one were in employment. The majority of participants used LSD to microdose, with one using both LSD and psilocybin.

**Table 2:** Participant overview

**Participant Pseudonym**	**Education**	**Occupation**	**Psychedelic used to microdose**
**Adam**	College	Working at a fast-food restaurant	Psilocybin
**Ben**	College	Engineer	Psilocybin
**Callum**	University	Audio engineer/musician	Psilocybin & LSD
**Daniel**	College	Online retail business owner	LSD
**Evan**	College/some university level courses	Customer service attendant in transport	Psilocybin
**Francesca**	Master’s degree	Research assistant/data scientist	LSD
**Gary**	Undergraduate degree	Hospitality	LSD
**Harris**	Master’s degree	IT systems manager	Psilocybin
**Isaac**	Undergraduate degree	IT systems admin	Psilocybin
**Jonah**	Undergraduate degree	UI/UX Designer	LSD
**Kim**	Undergraduate degree	Postgraduate student	LSD
**Leo**	School diploma and attended University but did not finish	Vice President of an engineering company	LSD
**Mia**	College and re-taking A Levels	Currently unemployed	LSD

### Procedure

Figure 1 details the procedure for this study.

### Conducting the interviews

In order for this study to be exploratory the interview was semi-structured (an interview schedule can be viewed in Additional files) so that the participants were not completely led by the questions and could discuss what was important to them [22].. It was noted that because the interviews would provide textual data (via the messenger) then embodied cognition (e.g. personal gestures and body language) could be missed from the data as compared to in-person interviews. This was overcome by the questions being designed in a way to gain a fuller understanding of participants' sense making. The interview commenced with a question which allowed the participant to give a descriptive account of their experience, with more analytical and narrative questions being asked once they had become more comfortable with the interview. Topics included reasons for microdosing, decisions to microdose, participants mental health and/or wellbeing experiences, and benefits and disadvantages to microdosing and the importance of these.

If it was felt that participants were over-disclosing during the interview (compromising their anonymity or discussing anything deemed incriminating such as how they buy illegal substances), then this was immediately brought to their attention and the interview paused so that these parts could be addressed and removed. Once the interview was completed, the data was saved using a researcher-generated identifier and a data key was created to enable participants to have their data deleted if requested during the two-week period following the interview. The key was deleted after the two weeks, and there was no record kept of the participant’s (unidentifiable) username, hence the link between participant username and interview data was irrevocably broken, meaning the data was rendered anonymous.

### Interpretative phenomenological analysis

IPA was chosen as a method to analyse the data as it is a phenomenological approach that explores how people make sense of their lived experiences. The approach is also informed by hermeneutics, in that the participant discusses their understanding of their experience, with the researcher then interpreting this to gain a deeper understanding of the experiential world of the participant. IPA is also idiographic, in that it seeks to understand the personal experience of the individual from their perspective. Hence, this approach was chosen as it facilitates a detailed exploration of the unique experience of the participants and how they make sense of microdosing psychedelic drugs [22]. IPA provided a complete framework for completing the research, compared to other methods such as Reflexive Thematic Analysis – an approach which is more flexible but less uniquely focussed on elucidating details of specific experiences.

Although this study gathered textual data, the interviews were completed live, so the interview was an interactive process between the interviewer and participant. Because of this interactive process, the researcher was still able to enter the lifeworld of the participant through the text, using a range a of questions from a semi-structured interview to achieve this. Hence, this approach offered a meticulous exploration of a phenomenon that required further, deeper understanding.

### Analysis

**Fig. 2:**
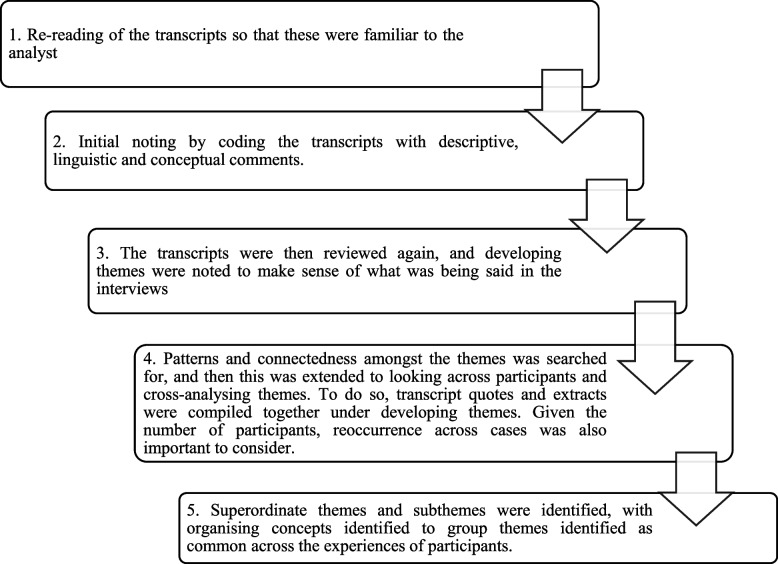
Process of IPA

As interviews were already typed, they did not require transcription. The IPA followed the process suggested by Smith et al. [22] which can be seen in Fig. 2.

In the process of IPA, the researcher was drawing upon the participants’ lived experience, trying to make sense of this, whilst also aware that this was being viewed through the researcher’s own lens (the ‘double hermeneutic’). This involved taking descriptions of participants experiences, considering the meaning that participants were attributing to these, and identifying the commonalities and differences between participants i.e., interpreting the descriptions provided by participants. In order to manage this interpretation of participants’ description of lived experiences, the researcher attended a regular IPA workshop group for the duration of the analysis, while supervision was used to highlight any biases. A reflective diary was kept by the researcher, so that any thoughts or judgements regarding the interviews could be considered in the analysis as a method to manage bias and support ‘bracketing’ [22].

## Results

The identified themes interpreted from the interviews were 1) Seeking a solution: Agency and rationale; 2) Microdosers as scientists; 3) Catalysing desirable and beneficial effects. All participants contributed to these themes.

### Theme 1: seeking a solution: agency and rationale

Microdosing psychedelic drugs was something that all participants had wondered about and considered whether it could be beneficial to themselves. All participants, except Leo, had been wanting to help themselves with an aspect of their lives in particular, whether it be mental health, relationships, cognition, or overall wellbeing. Some participants had reportedly been diagnosed with mental health illnesses, whilst others had self-diagnosed mental health illnesses and symptoms. There seemed to be an agency to their microdosing, in that they were making an active choice, and there was a sense of importance that this was not ‘just’ recreational drug use. In their agency, there also appeared to be a clear rationale for why they wanted to microdose.

Ben stated that he had been actively looking for something to support him with relationships, whereas others were looking for support with their mental health, such as Jonah who wanted support with anxiety and *“personal insecurities”.* Daniel described having a *“long-term anxiety disorder”* and depression, and was ‘self-medicating’ with alcohol and tobacco. Similarly, Evan “*had suffered from depression, ruminating thoughts, destructive thought-patterns and social anxiety… I have either just dealt with it or used, primarily alcohol, to deal with them…I wanted something that wasn't going to impair me”.*

As well as supporting with mental health, some participants talked about looking for additional support with their cognitive functioning. Kim mentioned turning to microdosing following a head injury as she had *“got to a point where I thought that if I had lost cognition, there was no point in living any more.”* Additionally, Callum added that creativity was something he had been looking for support with, which was in addition to sourcing support for *“mild depression”.*

There were five participants who spoke about how they had thought about, or sought, alternative methods of support. Jonah was considering seeing a therapist, whilst Daniel, Evan and Harris had tried conventional treatments for mental health prior to having microdosed. Harris stated that he had *“Multiple attempts at conventional treatment, therapy, self-help…Very little help from pharmaceuticals.”.* Mia spoke about her experience of getting support, explaining that she was “*in a pretty dark place with depression, anxiety, and substance abuse and couldn’t get professional help from [services] fast enough so I guess that was my easy escape*.”

Although Leo was not actively looking for support for a particular complaint, he still had a clear rationale for commencing microdosing. All participants described a justification for why they were doing an activity that was potentially illegal. There was a sense that having a rationale was important to the participants, and that microdosing had been carefully considered.

All participants except for Kim and Isaac had used psychedelic drugs in macrodoses previously. For Jonah and Adam, previous experience of drugs was part of the rationale for starting microdosing: This experience was not too dissimilar to that of Ben who stated *“I had taken a macro dose with an underground guide…And I thought it was very positive and was then much more interested in psychedelics.”* Daniel had also used psilocybin in the past and had used cannabis to help with his mental health. Isaac did not mention having used psychedelic drugs previously, but he did mention using cannabis. Across these participants, there was a sense that previous experience of drug use had been part of the rationale in choosing to microdose. For Gary, his experience of macrodosing had actually made him sceptical of microdosing, as he noted:

*“I was familiar with Macrodosing psychedelic substances, which made me a bit skeptical about the workings of microdosing…This made me want to see and feel the effects of microdosing for myself…Since the promised effects seemed beneficial for me”*. (Gary).

For all participants, there was a clear decision articulated to use microdosing and for some this was drawn from previous experiences of substance use. For all, the rationale for trying microdosing was to explore purported benefits of the approach.

### Theme 2: microdosers as scientists

In a manner seemingly related to the agency and rationale for microdosing, participants articulated how microdosing was not a haphazard decision for them. Indeed, it had been important for participants to carefully investigate microdosing, as they went about their microdosing journey in a regimented and scientific manner to inform their conclusions.

The majority of participants spoke about how they had conducted reading and research into microdosing, for example, reading a book by Michael Pollan like Ben and Evan, or articles and using online resources such as those found on Reddit. Callum spoke about his reading prior to microdosing, stating:

*“I approached it with knowledge rather than just for sake of it…I guess reading into psychedelics based on what was available back then and reading people’s use and reports…Really helped me to be better pilot…”.* (Callum).

There was a sense that for Callum his experience of microdosing was about being skilful and technical like a pilot, which enabled him to navigate and be in the driver’s seat of his experience of microdosing.

Although Gary and Adam did not explicitly state they had conducted any official research into microdosing, they both described watching YouTube videos on microdosing. Adam felt the idea of microdosing came *“to my head out of no where and i started doing it”,* aside from having conducted his own research by trying macrodoses of psilocybin. This was similar to Daniel, who had heard about microdosing through a friend, and applied the same *“logic”* as he had used for when self-medicating with cannabis prior to microdosing psilocybin.

Common across all participants was a language and description of experiences that was logical and scientific, creating a view of participants as experimenters in microdosing. Participants spoke about their ‘regime’ for microdosing, and as much as some participants had described copying a regime from elsewhere, they appeared to experiment with this so that it was tailored to their needs. Callum described starting his microdosing regime with the *“Hoffman schedule”*, but then created his own ‘schedule’ stating *“I was the rabbit in lab doing experiments on my brain”.* He completed experiments, for example *“I started with psilocybin and then included LSD just to see the differences which were vivid at least to me”*.

Although it was only half of participants who directly mentioned tolerance, most followed a regime such as a day on and a day off from microdosing. Isaac explained, *“I did Monday and Wednesday to help avoid any issues with tolerance and I didn't regularly need to feel the effects during the weekend.”* Adam was the only participant who seemed to follow a different regime, for which he had his own explicit reasoning, again highlighting how this was not ‘haphazard’ drug use.

Feeling the microdose was also apparent as all participants described a sensitivity to the immediate effects of the microdose. Participants explained that once they had microdosed there was an immediate effect that would last that day but would diminish over the non-microdosing days. Jonah summed this up stating *“I'd say once I get the benefits, they're permanently with me since it changes my outlook, which isn't a temporary thing. But the actual effects of the dose are gone after 12ish hours.”*

Evan was able to differentiate the effect of microdosing psilocybin compared to that of conventional treatments, adding that:

*“To me it's* [psilocybin] *usefulness is also tied to the fact that it is effects can be felt almost immediately compared to traditional pharmaceutical anti-depressants and even many holistic ones (e.g. St, Johns Wort).”* (Evan).

In terms of sensitivity, Adam, Ben, Kim and Leo all described a noticeable difference if they got their dosage of microdosing wrong, with Ben describing a *“fine line”* between taking a macro and a microdose. Again, this was indicative of a careful and regimented approach to microdosing, where effects were noted and changes to regimes implemented based on these.

There was a sense that participants were unable to come to solid conclusions regarding microdosing because they were logical regarding it, and did not jump to the conclusion that microdosing was a definite cure. It seemed important for participants to consider whether the effects of microdosing were permanent or not. Although opinion on this differed, it again felt considered and logical. Jonah, for example, acknowledged the impact of his previous experience with psychedelic drugs, explaining that microdosing:

*“Definitely had an impact, which is why I'm an advocate for the use and especially the research of it. I'd say a mix of microdosing and full trips, but definitely microdosing played a part. I'd say those benefits came from a 50/50 split.”* (Jonah).

Daniel noted other variables that positively impact on his mental health, and for Evan, although he attempted to separate the effects of microdosing from the other multiple supplements he was taking alongside, he noted “*I can not tell you with 100% certainty what the effectiveness of the other supplements are”.* Other participants added that microdosing could have led to healthier lifestyle behaviours, which then could have prolonged effects.

A number of participants were not shy of considering that microdosing could be a placebo effect. For Mia, there was a sense of her feeling particularly skilful and able from her microdosing experience. She explained:

*“I don't know if it was a placebo effect of genuinely the MD* [microdosing] *but I felt like a ninja…. I would love to credit it all to microdosing but I'm sure it's also partly my own awareness …I don't think I would've reached it on my own, however”* (Mia).

There was an impression that participants wished to share their findings. Because of their experiments and conclusions, it appeared important that they revealed their experience for the sake of others, even if microdosing could not be fully explained as a stand-alone cure. Daniel stated:

*“I really don't think it's a substance anywhere near as harmful as the current drug laws have it at in the world. Its a bit baffling when consider its low harm rate in comparison to. Other substances…I think it needs to be made much more easier to access and study for sensible adults”.* (Daniel).

Research in this field appeared important to participants, with Isaac stating* that “I’d much rather have a professional tell me the amount and how often I should take something but I am forced into doing my own research.*

The present theme of Microdosers as Scientists links to the previous theme, Agency and Rationale through the decisions made by participants to start microdosing and the way and manner in which participants approached this. For the participants, it seems that a logical and methodical approach to microdosing was described as they explored the potential effects on their wellbeing.

### Theme 3: catalysing desirable and beneficial effects

Participants conveyed a sense of understanding that microdosing was not a ‘cure’ for their difficulties. They spoke about how microdosing instead seemed to be a catalyst, or a tool, that enabled learning and elicited new behaviours that would then trigger and maintain any positive effects they happened to experience whilst microdosing.

Microdosing was described as something that was a *“buffer” and a “shield”* (Evan), a *“tool”* (Leo), and a *“jumpstart”* (Isaac), and a *“catalyst”* (Gary). Mia described that alongside microdosing she was able to *“train”* her brain to help her with anxiety, and added *“I would love to credit it all to microdosing but I'm sure it's also partly my own awareness …I don't think I would've reached it on my own.”.* In a similar sense, Harris explained* “It's not a magic bullet…the causes aren't solely biochemical so why would a biochemical treatment be the only thing required.”*

Callum talked about how microdosing helped him build a *“straight mind”*. He stated:

*“Microdosing made me really tap in, face my own “demons” and really work on myself. I was “juiced” lack of better word to start working out… I had to stop microdosing as I was traveling for six months…and I must say it was quite hard to keep straight mind. By straight mind I mean the mind that microdosing helped to built”.* (Callum).

For a few of the participants, this catalyst was described as something that discretely worked in the background, distinguishing this from recreational drug use. For example, for Harris “the point of *micro* dosing is for the effects to be largely *sub* liminal”.

All participants noted that there were perceived beneficial cognitive effects from microdosing. However, in the case of Evan this would be taken with caution, as he felt some supplements he was taking may have also contributed to better cognitive abilities. Possible cognitive benefits included thoughts not having such a hold over them, reduction of negative thoughts, being more mindful, ability to problem solve and generate ideas. A number of participants referred to improved focus and mental sharpness. Isaac explained that “*The best way to describe it was there was always so much noise going on in my head. Microdosing helped clear the noise and allow me to focus on things.”*

Although not an aim of this study, two participants spontaneously made mention of microdosing prompting exploration of the deeper mind. Callum described:

“It [microdosing] helped me get hold of my thoughts in same fashion meditation helps…And really dive deeper as far as I could to sort out things that I didn’t need anymore and basically understand myself from a fundamental stand point…In sense I was a psychologist to myself…Free consultation whenever unneeded :)…I needed*…Tapping into my own “demons” was a rewarding experience” (Callum).

In terms of consequences, Daniel mentioned a cognitive disadvantage of microdosing suggesting:

*“The only disadvantage I've had is there is no off switch as such on the mushrooms. If I take a microdose say 6pm instead of the morning. I may feel my brains more active say midnight still and I can't sleep because I'm thinking away more than usual”.* (Daniel).

Aside from Leo, all participants drew upon social benefits that they felt they experienced alongside microdosing. Such benefits included improved relationships, listening better, having more initiative or confidence, making more social effort and reduction in social anxiety. Kim summed up her benefits of microdosing, explaining:

“*I have gained so much confidence it's crazy. I have a new career lined up. I am sociable, I am happy to speak up for myself. I don't even recognise the person that I was!... No social anxiety…For the first time ever”.* (Kim)

As much as Kim mentioned social benefits, she was one participant to mention a *“mild”* disadvantage, stating *“Apart from being extra chatty on the days I take it, I can't think of any disadvantages.”* This was similar to Ben’s experience of being *“a bit too loud or exuberant in interactions”* when he takes too higher a dose.

Over half of participants spoke about the perceived physical benefits of microdosing. This was in terms of microdosing contributing to promotion of healthier behaviours, and reduction in other substance use (including alcohol) or the need for stimulants like coffee. Ben explained that *“I also notice less urges to use alcohol or other stimulants…Be it cannabis, snacking, *etc.*”.*

There was also a sense that there was a possible affective benefit to microdosing, with 10 of the participants discussing this. Participants touched upon improvements such as feeling more resistant to negativity, feeling more relaxed, less anxious, reduced depressive symptoms, improved mood, happier, a more positive outlook on life (towards themselves and others) and increased satisfaction. Mia also described the effect microdosing had on her symptoms of depression:

*“I don't know if you know this but when you're depressed your vision actually changes colour so you see life in more of a grayscale and when I microdosed that disappeared, everything was brighter again”* (Mia).

Mia explained that a disadvantage to microdosing can be that *“very very rarely if I get the type of physical anxiety* [physical sensations of anxiety] *it will feel worse than normal …but those instances are very rare”.* Gary also noted an affective disadvantage of microdosing, sometimes being “*overwhelmed by those powerful emotions…But that didn't happen often…It had both benefits and disadvantages”.* On a similar note, Jonah explained that:

*“I'd say on both full and microdoses the only disadvantage is being very sensitive to peoples energy's…Besides that, no other disadvantages from microdosing….In hindsight…I don't really know if being sensitive to energies/emotions is an advantage now that I'm thinking about it.”* (Jonah)

Although not mentioned by all participants, six participants did include creativity as a perceived benefit to microdosing. Callum had mentioned this in his reason for microdosing, but there was a sense that this was an added benefit to others.

There were some negative experiences that participants mentioned. Ben mentioned legal issues, which was similar to Evan who said *“Well, sometimes I do feel slightly hungover but that is rare and could also just be from stress but I feel it more acutely since I have been microdosing…. The fact that it is illegal is never far away.”* Although, Evan was not sure whether the hungover feeling could also be contributed to working shifts. Similar to Evan, Ben noted a “*slightly cloudy head”* could be a disadvantage, but this was in relation to him *“taking more than what I need”.* Taking too much of a dose, was something Adam and Leo also pointed out to be a disadvantage at times.

Overall, participants had a rationale for microdosing and had researched it, and it appeared that by microdosing they believed that they had achieved what they had set out to in terms of supporting their mental health and wellbeing, with microdosing being the catalyst to achieving this.

## Discussion

This study used Interpretive Phenomenological Analysis to explore the experiences of people who microdose psychedelic drugs to support their mental health and wellbeing. Three broad themes were interpreted from the interviews 1) Seeking a solution: Agency and rationale; 2) Microdosers as scientists; 3) Catalysing desirable and beneficial effects.

The aim of this study was to build upon previous research and provide clarification of findings using a novel methodology, analysis and a new sample. The results of this study developed and refined some outcomes of previous research as well as providing new insights into people’s experience of microdosing psychedelics. The findings of this research reveal that participants were approaching microdosing methodically and with purpose, with a clear aim in mind for what they wanted to achieve by microdosing. Whether participants were microdosing for mental health purposes such as support with depression, for cognitive purposes such as increased focus, or for social and creative purposes, for all participants there was a degree of agency and rationale to the practice of microdosing. The rationale for microdosing were similar to those reported by Hutten et al. [10], such as enhancement of mood and performance and symptom relief, but different to studies such as Prochazkova et al. [19], which focused more on creative-thinking, and Yanakieva et al. [27] whose study looked at time perception.

Similarly to some previous research into microdosing psychedelic drugs, participants reported perceived benefits to their overall sense of wellbeing and mental health including improved mood and reduction in anxiety and symptoms of depression. Participants also described experiencing improved focus, and being able to think more clearly. There was a reduction in social anxieties for a number of the participants which included improved relationships, feeling more confident, as well as improvements in listening and conversing with others. Over half the participants also reported perceived physical benefits of microdosing in promoting healthy behaviours and less temptation to use other substances. Although not all, some participants did mention creativity as a benefit to microdosing, which has also been an outcome of microdosing in other research [1, 2, 11].

Participants described seeking to better themselves in particular and controlled ways. This is in contrast to theories and research of drug seeking and drug taking behaviours, which highlight the loss of self-control or control impairment in such behaviours [16] or of inhibition dysfunction where control in substance use is impaired [26]. Some participants in this present study noted the limitations of psychedelic drugs if they were to take more than a microdose and described how too much of a dose could be undesirable. Hence, this conscious choice and differentiation of doses is quite different to starting to use substances and subsequently losing control or experience control impairment over their use.

The present study did not explore whether those who microdose are addicted to the substances they are using to microdose, however, there was a sense of this being a controlled behaviour with participants also talking about the ability to stop microdosing and explore whether the benefits still remained. The substances the participants were using to microdose have been reported to be relatively safe with no reported risk of dependence, with little documentation of adverse effects whilst using psychedelics recreationally [17]. It remains unclear whether the properties of the substance allow participants to navigate and control their experience in comparison to microdosing other substances that have a more addictive potential.

In the current study, participants reported using logic and research in their decision to microdose, and did not appear to microdose in a disorganised, unplanned or ‘out of control’ manner. An example of a theory that may explain such decision making and use of psychedelics is Rational Choice Theory, which refers to a process where an individual will weigh up the costs and the benefits of their behaviour [26]. Becker and Murphy [3] suggested that those who are addicted to particular substances or behaviours may actually consider the delayed effects of their addictive behaviour as well as immediate rewards. This theory highlights the importance of a forward-looking aspect in using psychoactive substances and which may suggest drug use can at times be more controlled.

All except one participant described how previous drug experience allowed them to make informed decisions, alongside research and exploration of the microdosing subject. However, even though this shows rationale and understanding prior to initiating a microdosing regime, it also brings into question whether high doses of psychedelic drugs that the participants had taken previously may have contributed to reported perceived changes in their wellbeing. Research by Robin Carhart-Harris has suggested that high doses of psilocybin were associated with remission of depressive symptomology for six months at follow-up [5]. Similarly, following the catalyst metaphor described in Theme 3, it could be questioned whether such macrodoses of psychedelics were the catalyst in subsequent psychological, behavioural and social changes that led to the effects participants described as linked to microdosing.

Similar to the study by Polito and Stevenson [18], participants in the present study shared experiences related to the longevity of effects, with some participants suggesting any benefit reduced over the following days. Some participants suggested that effects were lasting, and others added that the effects were lasting because microdosing acted as a catalyst that encouraged new healthy behaviours. However, unlike the Polito and Stevenson [18] study, the experience of participants in this study was that microdosing had helped them achieve what they initially aimed for in terms of their mental health and wellbeing.

### Strengths and limitations

As textual data was used as opposed to conducting the interviews in person, this means that non-verbal communication from participants was missing which may have limited participants ability to communicate their lived experience. However, text-based interviews may be less pressured, as participants can re-read the questions and consider their answers before responding. Given interviews were online, this meant that participants from all around the world could be recruited, although for the purposes of anonymity this data was not routinely gathered.

As participants in this study mostly provided a positive experience of microdosing, it is not clear whether these participants are representative of the wider microdosing community. It was also not clear what the participant’s intentions were in engaging in this study and sharing their positive experiences. This will need to be addressed in future research which seeks to explore potential negative experiences of microdosing.

From the descriptions of the participants in the present study, it appeared that all participants were of a similar level of education and clearly had the ability to access the internet. As such, the sample may not represent those who microdose but do not routinely use internet forums. This is a novel exploratory study in an important area of interest, with the purpose of starting to develop an understanding of some of the experiences of those who microdose, which can provide a platform to develop research with wider samples. During the analysis, to balance out any potential bias, the researcher’s claims were reviewed during formal peer-group and research supervision.

## Conclusions

It was apparent from this study that the individuals who microdose were approaching this practice methodically and with purpose. They were sensitive to the microdosing effects and were able to adapt and change their regime to suit their needs. They also had a clear aim in mind for what they wanted to achieve by microdosing and reported beneficial effects on their mental health, as well as social, physical and cognitive improvements. As participants had a clear rationale for microdosing and approached it in a scientific manner, there was a sense of this practice being different to more haphazard drug use. Participants experienced microdosing as a catalyst that enabled them to navigate them towards a healthier lifestyle.

Psychedelics research is still a developing and growing area of study. The findings of this study could offer direction for larger studies such as controlled studies where microdoses of psychedelics are administered to human participants. Following on from the discussion, future research could also explore whether there is any difference in the effects of those who have taken macrodoses of psychedelic drugs, versus those who have microdosed over a longer period of time.

It is important to establish whether there is an area of need that is being missed by current healthcare systems, which is leading people to microdose. Psychedelic drugs still remain illegal in most countries, so it is important to understand why people may be taking a risk in accessing them to try to support themselves. The present study has illuminated some of the participant experiences and processes around this area, suggesting that there are people who microdose psychedelics with a clear aim in mind to try to improve their mental health and wellbeing and they describe their use as particular and methodical. Future research can develop these initial insights by exploring the extent to which this is representative of wider samples including those who might have negative experiences of microdosing.

## Supplementary Information

**Additional file 1. **Interview Schedule. An example of questions asked to participants during the semi-structured interview.

## Data Availability

The datasets generated and/or analysed during the current study are not publicly available due for anonymity and confidentiality of the participants. Collation of themes and anonymised participants quotes are available from the corresponding author on reasonable request.

## Abbreviations

IPA: Interpretative Phenomenological Analysis
LSD: Lysergic acid diethylamide
