# Supplementary material for: Experiences of microdosing psychedelics in an attempt to support wellbeing and mental health
Source: BMC Psychiatry. 2023 Mar 14;23:160. doi: 10.1186/s12888-023-04628-9 (PMC10012542; doi:10.1186/s12888-023-04628-9)
Supplement: Supplementary file 1 — Additional file 1. Interview Schedule. An example of questions asked to participants during the semi-structured interview. [file 12888_2023_4628_MOESM1_ESM.docx]

**Additional File 1**

*Interview Schedule*

Example questions are as follows:

1. When did you first try microdosing, and what was that like?
2. Can you tell me about what classic psychedelic drugs you have used to microdose? How are you using them to microdose?
3. Currently what is it like to microdose these drugs? What have been your experiences of microdosing them?
4. Can you tell me your reasons for microdosing psychedelics?
5. I’d like to know some more about your decision to microdose psychedelics. Can you tell me how you came to use them? (If not already answered)
6. Can you tell me about your mental health/wellbeing experiences / experiences of feeling low / sad / anxiety (using their words which they have described).
7. How are things now you are using microdosing psychedelics? Are things the same/different?
8. Do you feel that microdosing has had any benefit or disadvantage to the psychological/wellbeing difficulties you described experiencing?
9. What do you think is the importance of these benefits or disadvantages?
10. How long do you notice any effect for?
11. Do you feel microdosing psychedelics has changed things for you in any particular way? Please tell me more about that.
